# News and Miscellany

**Published:** 1915-05

**Authors:** 


### News and Miscellany.

### Warning to Doctors.

A fraudulent concern which has been operating in New Orleans, with headquarters at the Maison Blanche Building, under the title of "The American Medical Index Company," or "Association," has already victimized many members of the profession through its agents in various parts of the country, especially the South and West. The fictitious company masqueraded in New Orleans as a medical reference or bibliographic bureau, which, for the small subscription of $5.00 or $10 per annum, would supply all subscribers, upon request, not only with abstracts, but entire reprints, of original articles, etc., of the medical literature of the world, in a very short time after publication. The pretensions of the company were preposterous and impossible, on their face, but by quoting the names of well known and respectable members of the profession as their endorsers and sponsors, and by other sundry misrepresentations, they succeeded in giving their scheme a *bona fide* appearance and in obtaining many subscriptions. The chief crook in the company, who styles himself Dr. A. C. Beck, absconded from New Orleans sometime in February, after defrauding several medical men, leaving his office rent and furniture unpaid. Since his departure, letters have been received from Mobile, Ala.; Nashville, Tenn.; Cincinnati, Columbus, Youngstown, Ohio; Louisville,

Ky., and other places, showing that the operations of Beck and his gang have been rapid and widespread.

The last letter received from Youngstown, Ohio, shows that Beck was last seen in that town on March 3d, where he succeeded in cashing a fictitious draft on New Orleans, on the strength of a bogus letter of credit purporting to have been signed by me, as one of the chief stockholders of the so-called American Medical Index Association. As a matter of fact, I have never seen Beck and know nothing of him except through his nefarious transactions.

A letter from Dr. W. S. Thorning, editor of the *South Texas Medical Record,* of Houston, Texas, published in the *Southern Medical Journal* of March, 1915, warns the readers of the *Journal* against the wiles of a certain "Jules Hanaford," who represented himself as the agent of the so-called "American Institute of Medical Research," of Indianapolis, Indiana. This party was either Beck himself, or a bird of the same feather. It is either that, in soliciting subscriptions, the same tactics have been adopted by the gang in various localities under different names. Not satisfied with defrauding these medical subscribers, they have robbed several innocent canvassers, chiefly young women, whom they engaged to solicit for them in different cities, far away from their homes. In some instances this Beck left these unfortunate victims stranded and penniless to shift for themselves, after absconding with their subscriptions, commissions, and even their personal funds, which Beck obtained by confidence methods, etc.

My interest in this matter has been roused chiefly through the numerous letters that I have received and from the unfortunate persons victimized by Beck and his gang, who have utilized my name to further their confidence game. I am, therefore, especially anxious that the editors of medical journals who may read this communication give timely warning to their readers of the dangers in the so-called "American Medical Index Association" and other concerns of the same ilk, which pretend to be domiciled in New Orleans, claiming any endorsement, approval, or association in any way, as this is not only a fraudulent concern, but a criminal organization, fit only for the consideration of the police.

(Original signed) R. MATAS,
2255 St. Charles Avenue,
New Orleans, La.

---

Doctor—"I am sorry to tell you, but this illness has left you a broken man."

Patient—"You bet I am, doc; I got your bill this morning."

